# Impact of Consumption of Specific Food Groups on Metabolic and Cardiovascular Disorders among Nurses: Framingham’s Multifactorial Predictive Model

**DOI:** 10.3390/jcm13185568

**Published:** 2024-09-19

**Authors:** Anna Bartosiewicz, Justyna Wyszyńska, Edyta Łuszczki, Anna Lewandowska, Małgorzata Zatorska-Zoła, Piotr Sulikowski, Piotr Matłosz

**Affiliations:** 1 Institute of Health Sciences, Medical College of Rzeszow University, 35-959 Rzeszow, Poland; jwyszynska@ur.edu.pl (J.W.); eluszczki@ur.edu.pl (E.Ł.); 2 Faculty of Healthcare, State Academy of Applied Sciences in Jaroslaw, 37-500 Jaroslaw, Poland; am.lewandowska@poczta.fm; 3 Independent, Public Healthcare Complex, John Paul II Municipal Hospital, 35-241 Rzeszow, Poland; mzola1@poczta.onet.pl; 4 Faculty of Computer Science and Information Technology, West Pomeranian University of Technology, 71-210 Szczecin, Poland; piotr.sulikowski@zut.edu.pl; 5 Institute of Physical Culture Science, Medical College of Rzeszow University, 35-959 Rzeszow, Poland; pmatlosz@ur.edu.pl

**Keywords:** dietary consumption, Framingham Risk Score, metabolic and cardiovascular disorders, nurses

## Abstract

**Objective:** This study aimed to analyze the relationship between the consumption of selected food products and the risk of prevalence of selected metabolic and cardiovascular disorders among nurses. **Methodology:** This cross-sectional study was conducted among 405 nurses. To achieve the study objective, body composition analysis (Tanita MC-980), blood pressure measurement (Welch Allyn 4200B), anthropometric measurements, lipid profile, fasting blood glucose (CardioChek PA), and surveys regarding the consumption of specific food groups were conducted. **Results:** More than half of the respondents were overweight or/and obese, and almost 40% had elevated blood pressure levels. The results obtained from logistic regression models indicated that the consumption of specific food product groups may predispose to/increase the risk of hypertension, abdominal obesity, overweight, obesity, body fat accumulation, and the risk of cardiovascular events. **Conclusions:** These findings highlight the importance of targeted nutritional strategies to enhance the health and professional efficacy of nursing staff, paving the way for improved healthcare practices.

## 1. Introduction

In recent years, with evolving trends in public health, there has been a growing interest in the impact of diet on various health indicators [1,2]. Numerous scientific studies have unequivocally shown that dietary habits significantly influence our health and well-being, and a proper diet plays a crucial role in preventing many chronic diseases and improving overall health status [2,3,4]. According to research by Willett and Stampfer et al., a well-balanced diet rich in diverse nutrients can substantially reduce the risk of developing conditions such as type 2 diabetes, cardiovascular diseases, certain types of cancer, and neurodegenerative diseases [5]. Regular consumption of vegetables, fruits, whole grains, low-fat proteins, and healthy fats is associated with lower cholesterol levels, better blood sugar regulation, and a reduced risk of many chronic diseases [6]. The Mediterranean and DASH diets, often cited as models of healthy eating, have been proven effective in enhancing cardiovascular health and longevity [7,8,9,10,11]. Previous studies have already emphasized that fruit and vegetable consumption may reduce the risk of coronary heart disease in part through the lowering of C-reactive protein (CRP) [12,13]. Esmaillzadeh et al. examined how fruit and vegetable consumption correlate with CRP levels and the occurrence of metabolic syndrome. Their research demonstrated that greater consumption of fruits and vegetables is linked to a reduced risk of metabolic syndrome, possibly due to decreased CRP levels. These results reinforce existing dietary guidelines that advocate for higher daily consumption of fruits and vegetables to prevent cardiovascular disease effectively [14].

The assessment of cardiovascular disease risk using the Framingham Risk Score is recognized as a crucial tool in cardiological prevention [15,16]. In studies conducted by Wilson et al., the use of this tool allowed for an effective prediction of cardiovascular events over the next ten years [15]. This study follows these findings, analyzing how the consumption of specific groups of products can impact the Framingham Risk Score and other key health indicators among nurses [16]. Nurses, as a professional group, represent a particularly interesting subject for study in this context, especially since numerous studies indicate that their health condition is worse compared to other professions [17,18]. Specific occupational demands and risks combined with irregular shift work significantly hinder proper and regular nutrition, thereby impacting the health of this professional group [19,20].

Given the critical role of nurses in society, there is an obligation for scientific entities to conduct research in this area to develop effective preventive and intervention strategies tailored to the specifics of nursing work [21]. It is particularly concerning that the life expectancy of Polish nurses is much shorter than that of the general population of Polish women [22]. 

Proper nutrition can help manage stress levels, improve concentration, and increase energy levels, which are essential for individuals working in such a demanding profession [23]. This study focusing on specific food groups provides valuable data on their impact on overall health and can contribute to a better understanding of the dietary needs of this professional group. The findings of such studies are expected to offer valuable insights for healthcare workers, dietitians, and policymakers, thereby contributing to enhanced work performance and overall well-being of nurses [23]. Furthermore, research indicates that health professionals who do not follow their own health advice are less likely to promote healthy lifestyle choices among their patients [24,25]. 

This study aimed to analyze the relationship between the consumption of selected food groups and the risk of prevalence of selected metabolic and cardiovascular disorders among nurses. 

To the best of our knowledge, this study is one of the first to evaluate how the consumption of selected food products affects the prevalence of metabolic and cardiovascular disorders among such an important occupational group. This study fills a gap in existing research by utilizing advanced measurement techniques and statistical analysis.

## 2. Materials and Methods

### 2.1. Study Participants

 This study was conducted in 2022 among 405 nurses working at a hospital in southeastern Poland, after obtaining the hospital director’s consent to conduct measurements. All measurements (body mass analysis, anthropometric assessments, lipid profile, fasting blood glucose, and blood pressure checks) were performed in the morning. Participation in the study was voluntary and without charge. The recruitment criteria included professionally active nurses who had no symptoms of infection in the last two weeks, who were not aware of any health issues, and who were willing to participate in the project. The exclusion criteria included individuals with a pacemaker or other electronic implants (due to the possibility that the current used in the study could interfere with the operation of these devices), pregnancy (as bioimpedance testing is generally not recommended for pregnant women because the effects of electrical current on the fetus have not been fully investigated), and individuals with metal implants (since metal can affect electrical conductivity in the body and impact the quality of the results). The age of the subjects was not a criterion for participation in the measurements. Data from the measurements of 405 nurses underwent statistical analysis. All measurements were performed by qualified personnel, after a short rest and after signing the consent to participate in the study.

When analyzing the data, the following criteria and cut-offs were adopted:

Body mass index (BMI): <17—severely underweight; 17–18.49—underweight; 18.5–24.99—normal body weight; 25–29.99—overweight; 30–34.99—first-degree obesity; 35–39.99—second-degree obesity; >40—third-degree obesity [26].

*Waist–hip ratio (WHR)* was calculated by dividing the waist circumference by the hip circumference. A score of 0.83 or higher in women and 0.96 or higher in men was considered to indicate an android body type. In contrast, a coefficient of 0.83 or less in women and 0.96 or less in men indicated a gynoid body type [27].

*Waist-to-height ratio (WHtR)*

Men and women:

A value of <0.5 indicates an index within normal limits;

Values in the range of 0.5–0.6 indicate increased cardiometabolic risk;

Values ≥0.6 indicate significantly increased cardiometabolic risk [28].

*Blood pressure*: optimal SBP <120 mmHg and DBP <80 mmHg; normal blood pressure 120–129 mmHg (SBP) and/or 80–84 mmHg (DBP); normal high pressure 130–139 mmHg (SBP) and/or 85–89 mmHg (DBP); grade 1 hypertension 140–159 mmHg (SBP) and/or 90–99 mmHg (DBP); grade 2 hypertension 160–179 mmHg (SBP) and/or 100–109 mmHg (DBP); grade 3 hypertension ≥180 mmHg (SBP) and/or ≥110 mmHg (DBP); isolated systolic hypertension ≥140 (SBP) and <90 mmHg (DBP) [29,30].

*Lipid profile*

TC: 150–190 mg/dL;

LDL cholesterol: less than 115 mg/dL;

HDL cholesterol: men above 40 mg/dL, women over 48 mg/dL;

TRG: below 150 mg/dL [31].

*Fasting glucose*

Less than 70 mg/dL—hypoglycemia;

Values 70 to 99 mg/dL—normal glucose level;

Values 100 to 125 mg/dL—elevated glucose levels—pre-diabetes;

Values ≥126 mg/dL in at least two measurements—diabetes mellitus [32].

*Body fat* (BFP—assessed using bioelectrical impedance analysis)

Body fat within normal limits: men: 10–25% fat tissue, women: 20–35% fat tissue;

Increased body fat: men: above 25% fat tissue, women: above 35% fat tissue;

Excessive fatness: men: above 30% fat tissue, women: above 40% fat tissue [33,34].

### 2.2. Questionnaire

Questionnaires were distributed in paper format, accompanied by an envelope to ensure that participants could submit their responses securely and confidentially. The survey gathered sociodemographic information and explored various health-related behaviors and conditions. It included questions about the respondents’ most commonly consumed food groups, salt consumption, participation in preventive health checks, weight management practices, smoking habits, work patterns, and self-rated health status. 

### 2.3. Framingham Risk Score 

The Framingham Risk Score (FRS) was utilized to assess cardiovascular risk among the study participants. This predictive model, derived from data collected in the Framingham Heart Study—a long-standing epidemiological study conducted in the United States—estimates the likelihood of experiencing a cardiovascular event within the next 10 years. The FRS calculation incorporates several established cardiovascular risk factors: age, gender, levels of HDL (high-density lipoprotein) and LDL (low-density lipoprotein) cholesterol, systolic blood pressure, smoking status, and diabetes status. Each factor contributes to a composite score that quantifies the overall cardiovascular risk for an individual [15,16].

The study methodology and the cut-off points used during the study have been published in detail in *BMC Public Health*, 2024 [35].

### 2.4. Statistical Analysis

The analysis was performed using the R program, version 4.2.1 [36]. The analysis of qualitative variables (i.e., not expressed in numbers) was performed by calculating the number and percentage of occurrences of each value. Single- and multifactor analyses were performed using logistic regression. The results are presented as values of odds ratio (OR) parameters with a 95% confidence interval. The variables for the multivariate analysis were selected based on their significance in the one-factor analyses. 

The EPV (events per variable) index for the analysis was as follows: hypertension = 16.1; abdominal obesity according to WHR = 21.9; cardiometabolic risk according to WHtR = 14.8; overweight according to BMI = 13.75; obesity according to BMI = 10.8; increased amount of adipose tissue = 21.5; Framingham Risk Score = 10.6.

The quality of the multivariate models was assessed using the ROC (receiver operating characteristic) curves and the areas under the curve (AUCs). The analysis adopted a significance level of 0.05. Thus, all *p*-values below 0.05 were interpreted as significant associations. 

## 3. Results

A total of 405 nurses took part in the measurements, with a significant majority being women (*n* = 380; 93.83%). The average age of the participants was about 48.5 years. Detailed characteristics of the study group are shown in Table 1.

The reported daily consumption rates for specific food product groups are as follows: white bread 56.3%, dark bread 25.4%, fish and seafood 1.7%, red meat and sausages 19.2%, sour milk products 27.9%, cheese 25.1%, cottage cheese 22.2%, fruits and vegetables 70.1%, sweets and salty snacks 27.4%, and fast food products 4.6%. Detailed frequencies for the consumption of selected food product groups are presented in Table 2.

The results obtained from logistic regression models indicated that the consumption of specific food product groups may predispose to/increase the risk of hypertension, abdominal obesity, overweight, obesity, body fat accumulation, and the risk of cardiovascular events. The adjusted regression models revealed that significant predictors for the prevalence of abdominal obesity according to WHR were male gender (OR = 0.317) and age (OR = 1.087), while for cardiometabolic risk according to WHtR, the predictors were male gender (OR = 5.082), age (OR = 1.099), sweetening coffee/tea (OR = 1.614), and consuming red meat and sausages 2–4 times a week (OR = 1.898). Significant predictors for the prevalence of overweight were age (OR = 1.066), consuming fish and seafood a few times a month (OR = 0.44), and consuming red meat and sausages 2–4 times a week (OR = 2.208), while for obesity, they were age (OR = 1.095) and sweetening coffee/tea (OR = 1.978). In the case of increased body fat accumulation and the risk of cardiovascular events, significant predictors were male gender (OR = 3.296; OR = 126.311) and age (OR = 1.093; OR = 1.751), (Table 3). 

Details of univariate and multivariate regression analyses are available in the Supplementary Materials Tables S1–S7.

**Figure 1:**
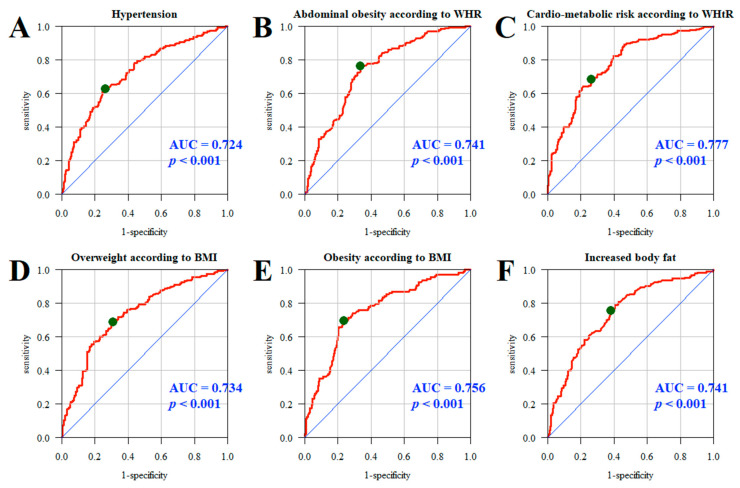
ROC curve and AUC value: hypertension (**A**), abdominal obesity according to WHR (**B**), cardiometabolic risk according to WHtR (**C**), overweight (**D**), obesity (**E**), and increased body fat (**F**). Line red—ROC curve for the analyzed model. Line blue—ROC curve for the random model (depicting a line with a 45-degree slope, indicating no predictive ability). Green dot—optimal cutpoint on the red curve, which is the point closest to the top-left corner of the plot, representing the best trade-off between sensitivity and specificity.

Additionally, the quality and effectiveness of multifactorial models in predicting the impact of specific product groups on selected health indicators and the Framingham Risk Score were assessed in the study group of nurses. ROC curves and AUCs for models A–F showed good predictive accuracy for hypertension (AUC = 0.724, *p* < 0.001); abdominal obesity measured by WHR (AUC = 0.741, *p* < 0.001); cardiometabolic risk according to WHtR (AUC = 0.777, *p* < 0.001); overweight (AUC = 0.734, *p* < 0.001) and obesity (AUC = 0.756, *p* < 0.001) determined by BMI; and excessive body fat (AUC = 0.741, *p* < 0.001) (Figure 1).

**Figure 2:**
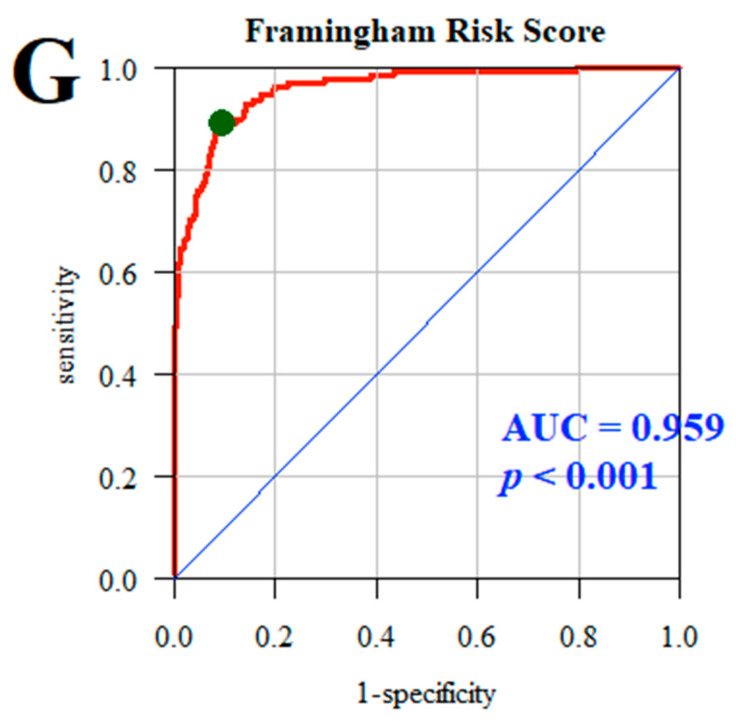
Likelihood of experiencing a cardiovascular event within the next 10 years according to Framingham Risk Score. Line red—ROC curve for the analyzed model. Line blue—ROC curve for the random model (depicting a line with a 45-degree slope, indicating no predictive ability). Green dot—optimal cutpoint on the red curve, which is the point closest to the top-left corner of the plot, representing the best trade-off between sensitivity and specificity.

In the case of model G, which predicted the Framingham Risk Score, which estimates the likelihood of experiencing a cardiovascular event within the next 10 years, the AUC was 0.959, *p* < 0.001, indicating that the multifactorial model almost perfectly predicts cardiovascular risk (Figure 2). 

This study demonstrated that the diet of nurses significantly impacts the risk of developing metabolic and cardiovascular disorders, with clear associations between specific food groups and health outcomes such as abdominal obesity, overweight, and cardiometabolic risk. Multifactorial modeling using the Framingham Risk Score effectively predicts cardiovascular risk (AUC = 0.959), confirming the potential of diet as a critical tool in health prevention among this professional group.

## 4. Discussion

This study demonstrates the influence of dietary patterns on metabolic and cardiovascular disorders among 405 predominantly female nurses, with a mean age of 48.5 years. Consumption data show a significant preference for fruits and vegetables (70.1%) and white bread (56.3%), consumed every day, while fish and seafood were the least consumed (1.7%). Logistic regression analyses highlighted significant predictors of health risks, such as hypertension, obesity, and cardiovascular events, to be notably male gender, increased age, and the consumption of red meat and sweetening coffee/tea. Importantly, the models exhibited strong predictive accuracy for cardiovascular risk, with an AUC of 0.959 for the Framingham Risk Score. These findings reinforce a significant link between dietary choices and health outcomes, emphasizing the need for targeted nutritional interventions to mitigate diet-related risks and promote overall health among nurses, who must be aware of the impact of their dietary habits impact of their health and well-being. The Global Burden of Disease study underscores the profound influence of diet on the incidence of major health conditions [37]. Despite guidance from health organizations like The European Society of Cardiology, adherence to dietary recommendations remains low, contributing to cardiovascular disease being a leading cause of death in Europe. This trend is exacerbated by inadequate nutritional knowledge and the influence of unhealthy dietary patterns increasingly adopted globally [38,39,40]. Nurses are pivotal in promoting healthy lifestyles and reducing cardiovascular risk [41]; however, their nutritional knowledge is often insufficient. A study involving 506 nurses revealed that only 58.4%, correctly understood nutrition related to obesity and cardiovascular disease [41,42]. Despite recognizing the benefits of certain nutrients, many lacked detailed knowledge about low-cholesterol diets and sources of water-soluble fiber, fatty acids, and specific cardioprotective foods [43].

Warber et al. observed a 66% accuracy rate in nutritional knowledge among nurse practitioners, underscoring the need to promote current, evidence-based nutrition education. This knowledge enhances their capacity to educate patients and integrate healthy practices into their own lives [44]. A study conducted by Kilar et al. found that 60% of nurses engaged in preventive examinations, and 70% prioritized family with health and work following closely. However, nurses with chronic conditions had lower health behavior levels than their healthy counterparts, highlighting the need for targeted health interventions within this group [45]. The findings of this study highlight the need for specific interventions to improve the health of nurses with chronic illnesses and to promote a more proactive approach to personal health management within the nursing profession. Research on the association between dietary patterns and the risk of metabolic syndrome or cardiovascular disease among nurses is limited and inconsistent. A study of 346 pre-registered UK nurses and midwives found a high prevalence of overweight or obese (33.8%) and poor dietary habits, with 67.6% not meeting the five-a-day fruit and vegetable guideline. Such participants were also less likely to view health professionals as role models, associating negative perceptions with health promotion [46]. Similarly, another study showed that over 50% of nurses did not meet recommended physical activity levels, and more than 75% did not consume adequate amounts of fruits and vegetables, with nearly 20% reporting current smoking habits [47]. Our study revealed a higher prevalence of smoking among nurses, with 24.6% reporting this habit. A significant majority of respondents (70.1%) indicated a daily consumption of fruits and vegetables; however, the survey did not assess whether they met the recommended intake of five servings per day. In contrast, studies in different regions like Australia and Brazil show varying adherence to dietary guidelines among nurses, indicating a complex relationship between professional health knowledge and personal health practices [48,49].

A nutritious diet is crucial for overall well-being and longevity; however, many nurses struggle to maintain healthy eating habits despite their role as advocates for health education. Research indicates a significant and growing trend of overweight and obesity among nurses, with a prevalence study in Scotland revealing that 69% of nurses were overweight or obese—rates notably higher than other healthcare professionals and those in non-health-related jobs [50,51]. In our study, the prevalence of excess weight based on BMI was nearly 60%, with 32.6% of participants classified as having obesity. Significant predictors for the prevalence of obesity included age (OR = 1.095) and the practice of sweetening coffee or tea (OR = 1.978). This trend may be linked to low physical activity and diets deficient in fruits and vegetables but high in sugar, exacerbated by shift work [52,53,54]. Although no single intervention has shown strong effectiveness against obesity, integrating tailored lifestyle interventions into nurses’ routines is essential for addressing their unique challenges in maintaining a healthy lifestyle [55].

A study of 403 female nurses examined the links between dietary habits, alcohol use, and shift work on the risk of metabolic syndrome, finding a prevalence of 5.6%. The key risk factors included late-night calorie intake, carbonated drink consumption, family history of diabetes, and non-shift work [56]. Conversely, a cross-sectional study of 420 female Iranian nurses found a metabolic syndrome prevalence of 3.6%, with no significant correlations to waist circumference, blood pressure, triglycerides, HDL cholesterol, or fasting blood sugar, after controlling confounding factors [57]. A global systematic review and meta-analysis of 22 articles encompassing 117,922 nurses identified a sedentary lifestyle and lack of physical activity as the most prevalent cardiovascular disease risk factors, observed in 46.3% of participants. Additional risk factors included a family history of cardiovascular disease (41.9%), being overweight (33.3%), and alcohol consumption (24.6%). Among shift-working nurses, nearly all risk factors were more pronounced, suggesting worse conditions compared to their daytime counterparts [58]. These findings highlight the need for targeted interventions to mitigate cardiovascular disease risk among nurses, particularly those in shift work environments.

Future research should explore interventions aimed at improving dietary quality and increasing physical activity among nurses, assessing their long-term impact on health outcomes. Universities must prioritize health promotion initiatives, improving access to resources for nursing students and staff to foster a culture of well-being and resilience that enhances educational outcomes and patient care. The key initiatives could include promoting regular rest breaks, providing on-site fruits and vegetables, and incorporating brief exercise sessions into daily routines. Educational programs need to emphasize workplace well-being and self-care, addressing both personal and environmental factors affecting health-related decisions.

### Strengths, Limitations, and Future Research

Based on our understanding, this study represents one of the earliest comprehensive efforts in Poland to explore how consumption of certain food groups affects the risk of prevalent metabolic and cardiovascular disorders within this professional group. It is important to acknowledge several potential limitations of this study that may affect the interpretation of its findings. The study’s geographical reach was restricted and should be expanded to include more medical facilities across different regions. Given its cross-sectional design, it is inappropriate to infer causality or temporal relationships from the results. Additionally, some variables, such as alcohol consumption, physical activity, and menopausal and hormonal status, were not included in the selection criteria, although we acknowledge their potential impact on health. Further research involving a larger population that considers age-specific factors and other lifestyle variables is necessary.

## 5. Conclusions

This study reveals significant relationships between dietary habits and various health risks, particularly related to hypertension, obesity, and cardiovascular events. The key findings indicate that the consumption of certain food groups, particularly red meat and processed meats like sausages, 2 to 4 times per week is associated with an elevated prevalence of overweight and cardiometabolic risk. Factors such as male gender and age were significantly associated with abdominal obesity and cardiometabolic risk. Age was also a significant factor in the prevalence of overweight and obesity; moreover, sweetening coffee/tea was identified as a significant factor associated with obesity and cardiometabolic risk. These results underline the importance of dietary patterns in managing health outcomes among nurses.

## Figures and Tables

**Table 1:** Characteristics of the study group [35].

Variable		Total (*n* = 405) *n* (%)
Sex *	Female	380 (93.8)
	Male	25 (6.1)
Age (years)	Average ± SD	48.4 (10.37)
	Median (quartiles)	51 (42–55)
Place of residence *	City	209 (51.6)
	Village	196 (48.4)
Type of work *	Staff management/administration	60 (14.8)
	Hospital ward	344 (85.1)
Work system *	One shift work	140 (34.5)
	Shift work and night duty	265 (65.4)
More than one job *	No	238 (58.7)
	Yes	167 (41.2)
Education *	Basic nursing education	131 (32.3)
	Bachelor	95 (23.4)
	Master’s degree	179 (44.2)
Participation in preventive examinations other than obligatory *	No	293 (72.3)
	Yes	112 (27.6)
Cigarettes smoking *	No	305 (75.3)
	Yes	100 (24.6)
Adding sugar to coffee/tea *	No	175 (43.2)
	Yes	230 (56.7)
Salting dishes *	I do not use salt at all	6 (1.4)
	Rarely or never add salt to food	71 (17.5)
	I taste the food and add salt as needed	234 (57.7)
	I add salt to my food without trying it first	94 (23.2)
Weight self-control *	Every day	16 (3.9)
	Once a week	69 (17.0)
	Twice a week	46 (11.3)
	Once a month	100 (24.6)
	Hardly ever	72 (17.7)
	I do not check my weight regularly	102 (25.1)
Self-assessment of the material situation *	Very good	30 (7.4)
	Good	208 (51.3)
	Average	158 (39.0)
	Bad	9 (22.2)
BMI * (kg/m^2^)	Underweight	7 (1.7)
	Normal body mass	158 (39.0)
	Overweight	132 (32.6)
	Obesity	108 (26.7)
	Median (quartiles)	26.2 (23–30)
WHR *	Normal	208 (51.4)
	Abdominal obesity	197 (48.6)
	Median (quartiles)	0.83 (0.78–0.88)
WHtR *	Normal	178 (43.9)
	Increased cardiometabolic risk	164 (40.5)
	Significantly increased cardiometabolic risk	63 (15.6)
	Median (quartiles)	0.51 (0.46–0.57)
BP * (mmHg)	Normal	173 (42.7)
	Elevated	71 (17.5)
	Hypertension	161 (39.8)
	SBP Median (quartiles)	121.6 (110–132)
	DBP Median (quartiles)	75.6 (69–81)
BFP category * (%)	Normal	233 (57.5)
	Elevated	172 (42.5)
	Median (quartiles)	31.9 (26–36.8)
FG * (mg/dL)	Normal	276 (68.1)
	Elevated	123 (30.4)
	Abnormal glucose—suspicion of diabetes	6 (1.5)
	Median (quartiles)	97 (90–102)
HDL * (mg/dL)	Decreased	94 (23.2)
	Normal	311 (76.8)
	Median (quartiles)	57 (49–66)
LDL * (mg/dL)	Elevated	207 (51.1)
	Normal	198 (48.9)
	Median (quartiles)	117 (88–144)

Data presented as follows: *—n (%); BMI—body mass index; WHR—waist–hip ratio; WHtR—waist-to-height ratio; BP—blood pressure; SBP—systolic blood pressure; DBP—diastolic blood pressure; BFP category—body fat percentage category; LDL—low-density lipoprotein cholesterol; HDL—high-density lipoprotein cholesterol; FG—fasting glucose.

**Table 2:** Consumption frequency of specific groups of products [35].

Group of Products	Frequency of Consumption				
	I Don’t Eat *n* (%)	A Few Times a Month *n* (%)	2–4 Times a Week *n* (%)	Once a Week *n* (%)	Every Day *n* (%)
White bread	36 (8.8)	45 (11.1)	49 (12.1)	47 (11.6)	228 (56.3)
Wholemeal bread	66 (16.3)	69 (17.0)	88 (21.7)	79 (19.5)	103 (25.4)
Fishes and seafood	60 (14.8)	193 (47.6)	35 (8.6)	110 (27.1)	7 (1.7)
Red meat, ham, sausages	35 (8.6)	111 (27.4)	117 (28,8)	64 (15.8)	78 (19.2)
Sour milk products	44 (10.8)	50 (12.3)	119 (29.3)	79 (19.5)	113 (27.9)
Cheese	22 (5.4)	114 (28.1)	85 (20.9)	82 (20.2)	102 (25.1)
Cottage cheese	12 (2.9)	73 (18.0)	141 (34.8)	89 (21.9)	90 (22.2)
Vegetables/fruit	3 (0.7)	11 (2.7)	76 (18.7)	31 (7.6)	284 (70.1)
Sweets/salty snacks	13 (3.2)	65 (16.0)	132 (32.5)	84 (20.7)	111 (27.4)
Fast food products	168 (41.4)	159 (39.2)	28 (6.9)	31 (7.6)	19 (4.6)

**Table 3:** Significant predictors of the odds of hypertension, abdominal obesity, cardiometabolic risk, overweight, obesity, increased body fat, and cardiovascular risk—univariate and multiple regression models.

Variable		Parameter **							
		Univariate Model				Multiple Model			
		OR	95% CI		*p*	OR	95% CI		*p*
Consumption of specific groups of products and the prevalence of hypertension									
Age	(ears)	1.068	1.043	1.093	<0.001 *	1.072	1.047	1.098	<0.001 *
Salting dishes	Rarely or never add salt to food	1	ref.			1	ref.		
	I taste the food and add salt as needed	0.58	0.344	0.98	0.042 *	0.528	0.299	0.93	0.027 *
	I add salt to my food without trying it first	1.091	0.597	1.995	0.776	1.089	0.553	2.141	0.806
Consumption of specific groups of products and the prevalence of abdominal obesity according to WHR									
Sex	Female	1	ref.			1	ref.		
	Male	0.312	0.122	0.8	0.015 *	0.317	0.116	0.864	0.025 *
Age	(years)	1.089	1.063	1.115	<0.001 *	1.087	1.061	1.115	<0.001 *
Consumption of specific groups of products and the prevalence of cardiometabolic risk according to WHtR									
Sex	Female	1	ref.			1	ref.		
	Male	2.619	1.023	6.702	0.045 *	5.082	1.583	16.319	0.006 *
Age	(years)	1.095	1.07	1.122	<0.001 *	1.099	1.071	1.127	<0.001 *
Adding sugar to coffee/tea	No	1	ref.			1	ref.		
	Yes	1.449	0.975	2.155	0.067	1.614	1.013	2.571	0.044 *
Red meat, ham, sausages	A few times a month or less	1	ref.			1	ref.		
	2–4 times a week	1.682	1.082	2.614	0.021 *	1.898	1.135	3.174	0.015 *
	Every day	1.646	0.942	2.875	0.08	1.556	0.823	2.944	0.174
Consumption of specific groups of products and the prevalence of overweight according to BMI									
Age	(years)	1.067	1.044	1.089	<0.001 *	1.066	1.042	1.091	<0.001 *
Fishes and seafood	I do not eat	1	ref.			1	ref.		
	A few times a month	0.442	0.238	0.822	0.01 *	0.44	0.219	0.882	0.021 *
	Once a week	0.824	0.432	1.572	0.557	0.597	0.284	1.255	0.174
Red meat, ham, sausages	A few times a month or less	1	ref.			1	ref.		
	2–4 times a week	2.184	1.394	3.423	0.001 *	2.208	1.347	3.618	0.002 *
	Every day	1.602	0.917	2.797	0.098	1.447	0.776	2.698	0.45
Consumption of specific groups of products and the prevalence of obesity according to BMI									
Age	(years)	1.091	1.06	1.124	<0.001 *	1.095	1.061	1.129	<0.001 *
Adding sugar to coffee/tea	No	1	ref.			1	ref.		
	Yes	2.082	1.303	3.326	0.002 *	1.978	1.19	3.289	0.009 *
Consumption of specific groups of products and the prevalence of increased body fat									
Sex	Female	1	ref.			1	ref.		
	Male	2.131	0.933	4.866	0.073	3.296	1.281	8.478	0.013 *
Age	(years)	1.088	1.061	1.115	<0.001 *	1.093	1.065	1.121	<0.001 *
Consumption of specific groups of products and the prevalence of and increased cardiovascular risk—Framingham Risk Score									
Sex	Female	1	ref.			1	ref.		
	Male	3.134	1.369	7.177	0.007 *	126.311	11.981	1331.671	<0.001 *
Age	(years)	1.623	1.455	1.811	<0.001 *	1.751	1.535	1.997	<0.001 *

* Statistically significant relationship (*p* < 0.05); *p*—single and multivariate linear regression; OR—odds ratio; CI—confidence interval; OR (95% CI)—odds ratio with a 95% confidence interval; ** adjusted to white bread, wholemeal bread, sour milk products, cheese, cottage cheese, vegetables/fruit, sweets/salty snacks, and fast food products.

## Data Availability

All the data generated or analyzed during this study are included in this published article.

## Supplementary Materials

The following supporting information can be downloaded at: https://www.mdpi.com/article/10.3390/jcm13185568/s1, Table S1: Consumption of specific food groups and prevalence of hypertension. Univariate and multiple regression models. Table S2: Consumption of specific food groups and prevalence of abdominal obesity according to WHR. Univariate and multiple regression models. Table S3: Consumption of specific group of products and the prevalence of cardiometabolic risk according to WHtR. Univariate and multiple regression models. Table S4: Consumption of specific group of products and the prevalence of overweight according to BMI. Univariate and multiple regression models. Table S5: Consumption of specific group of products and the prevalence of obesity according to BMI. Univariate and multiple regression models. Table S6: Consumption of specific group of products and the prevalence of increased body fat. Univariate and multiple regression models. Table S7: Consumption of specific group of products and the prevalence of and increased cardiovascular risk-Framingham Risc Score. Univariate and multiple regression models.
