# Supplementary material for: Impact of Consumption of Specific Food Groups on Metabolic and Cardiovascular Disorders among Nurses: Framingham’s Multifactorial Predictive Model
Source: J Clin Med. 2024 Sep 19;13(18):5568. doi: 10.3390/jcm13185568 (PMC11432469; doi:10.3390/jcm13185568)
Supplement: Supplementary file 1 [file jcm-13-05568-s001.zip › jcm-3192547-supplementary.pdf]

Supplementary materials - tables from 1 to 7 [S1 – S7]

Table S-1. Consumption of specific food groups and prevalence of hypertension. Univariate and multiple regression models.

Variable		Univariate model				Multiple model			
		OR	95%CI		p	OR	95%CI		p
Sex	Female	1	ref.			1	ref.		
	Male	1.698	0.755	3.822	0.201	1.931	0.785	4.751	0.152
Age	[years]	1.068	1.043	1.093	<0.001 *	1.072	1.047	1.098	<0.001 *
Adding sugar to coffe/tea	No	1	ref.			1	ref.		
	Yes	1.377	0.919	2.065	0.121	1.327	0.844	2.086	0.22
Salting dishes	Rarely or never add salt to food	1	ref.			1	ref.		
	I taste the food and add salt as needed	0.58	0.344	0.98	0.042 *	0.528	0.299	0.93	0,027 *
	I add salt to my food without trying it first	1.091	0.597	1.995	0.776	1.089	0.553	2.141	0.806
White bread	Every day	1	ref.						
	Not every day	0.832	0.557	1.245	0.372				
Wholemeal bread	A few times a month or less	1	ref.			1	ref.		
	2-4 times a week	0.701	0.441	1.115	0.133	0.684	0.412	1.137	0.143
	Every day	0.827	0.491	1.391	0.473	0.865	0.484	1.547	0.626
Fishes and seafood	I do not eat	1	ref.						
	A few times a month	1.115	0.615	2.02	0.72				
	One’s a week or more often	1.021	0.552	1.886	0.948				
Red meat, ham, sausages	A few times a month or less	1	ref.						
	2-4 times a week	1.062	0.679	1.66	0.793				
	Every day	1.179	0.674	2.062	0.565				
Sour milk products	A few times a month or less	1	ref.						

Variable		Univariate model				Multiple model			
		OR	95%CI		p	OR	95%CI		p
	2-4 times a week	0.841	0.51	1.385	0.497				
	Every day	0.893	0.512	1.558	0.691				
Cheese	A few times a month or less	1	ref.			1	ref.		
	2-4 times a week	1.544	0.968	2.462	0.068	1.649	0.993	2.74	0.053
	Every day	1.172	0.688	1.998	0.559	0.992	0.557	1.768	0.979
Cottage cheese	A few times a month or less	1	ref.						
	2-4 times a week	0.837	0.504	1.392	0.494				
	Every day	1.195	0.657	2.176	0.559				
Vegetables/fruit	Every day	1	ref.						
	Not every day	1.152	0.748	1.776	0.52				
Sweets/salty snacks	A few times a month or less	1	ref.						
	2-4 times a week	0.761	0.45	1288	0.309				
	Every day	0.95	0.53	1.706	0.865				
Fast food products	I do not eat	1	ref.						
	I eat	1.227	0.817	1.841	0.324				

* Statistically significant relationship ($p<0.05$); *p* – single and multivariate linear regression; OR - odds ratio; CI – confidence interval; OR (95% CI) - odds ratio with a 95% confidence interval.

Table S-2. Consumption of specific food groups and prevalence of abdominal obesity according to WHR. Univariate and multiple regression models.

Variable		Univariate model				Multiple model			
		OR	95%CI		p	OR	95%CI		p
Sex	Female	1	ref.			1	ref.		

Variable		Univariate model				Multiple model			
		OR	95%CI		p	OR	95%CI		p
	Male	0.312	0.122	0.8	0.015 *	0.317	0.116	0.864	0.025 *
Age	[years]	1.089	1.063	1.115	<0.001 *	1.087	1.061	1.115	<0.001 *
Adding sugar to coffe/tea	No	1	ref.						
	Yes	0.965	0.651	1.431	0.86				
Salting dishes	Rarely or never add salt to food	1	ref.						
	I taste the food and add salt as needed	0.835	0.499	1.398	0.492				
	I add salt to my food without trying it first	0.886	0.485	1.62	0.695				
White bread	Every day	1	ref.						
	Not every day	0.883	0.596	1.308	0.535				
Wholemeal bread	A few times a month or less	1	ref.			1	ref.		
	2-4 times a week	1.344	0.851	2.12	0.205	1.359	0.81	2.282	0.246
	Every day	1.521	0.908	2.548	0.111	1.399	0.775	2527	0.266
Fishes and seafood	I do not eat	1	ref.			1	ref.		
	A few times a month	0.641	0.357	1.149	0.135	0.656	0.337	1.277	0.215
	One’s a week or more often	0.745	0.408	1.359	0.337	0.601	0.299	1.207	0.153
Red meat, ham, sausages	A few times a month or less	1	ref.						
	2-4 times a week	1.471	0.949	2.28	0.084				
	Every day	0.963	0.553	1.676	0.894				
Sour milk products	A few times a month or less	1	ref.						
	2-4 times a week	0.886	0.542	1.448	0.628				
	Every day	1.018	0.589	1.759	0.949				

Variable		Univariate model				Multiple model			
		OR	95%CI		p	OR	95%CI		p
Cheese	A few times a month or less	1	ref.						
	2-4 times a week	1.166	0.741	1.835	0.506				
	Every day	1	0.598	1.673	1				
Cottage cheese	A few times a month or less	1	ref.			1	ref.		
	2-4 times a week	1.071	0.649	1.768	0.789	1.099	0.63	1.919	0.739
	Every day	2.137	1.167	3.911	0.014 *	1.703	0.864	3.355	0.124
Vegetables/fruit	Every day	1	ref.						
	Not every day	0.874	0.57	1.338	0.535				
Sweets/salty snacks	A few times a month or less	1	ref.						
	2-4 times a week	1.108	0.66	1.861	0.698				
	Every day	0.977	0.547	1.744	0.936				
Fast food products	I do not eat	1	ref.						
	I eat	0.875	0.589	1.3	0.508				

* Statistically significant relationship (p<0.05); *p* – single and multivariate linear regression; OR - odds ratio; CI – confidence interval; OR (95% CI) - odds ratio with a 95% confidence interval; WHR - Waist Hip Ratio.

Table S-3. Consumption of specific group of products and the prevalence of cardiometabolic risk according to WHtR. Univariate and multiple regression models.

Variable		Univariate model				Multiple model			
		OR	95%CI		p	OR	95%CI		p
Sex	Female	1	ref.			1	ref.		

Variable		Univariate model				Multiple model			
		OR	95%CI		p	OR	95%CI		p
	Male	2.619	1.023	6.702	045 *	5.082	1.583	16.319	0.006 *
Age	[years]	1.095	1.07	1.122	<0.001 *	1.099	1.071	1.127	<0.001 *
Adding sugar to coffe/tea	No	1	ref.			1	ref.		
	Yes	1.449	0.975	2.155	0.067	1.614	1.013	2.571	0.044 *
Salting dishes	Rarely or never add salt to food	1	ref.						
	I taste the food and add salt as needed	1.33	0.793	2.231	0.28				
	I add salt to my food without trying it first	1.007	0.551	1.841	0.981				
White bread	Every day	1	ref.						
	Not every day	0.914	0.616	1.357	0.656				
Wholemeal bread	A few times a month or less	1	ref.			1	ref.		
	2-4 times a week	1.167	0.74	1.84	0.506	1.386	0.805	2.389	0.239
	Every day	1.524	0.904	2.568	0.114	1.702	0.894	3.239	0.105
Fishes and seafood	I do not eat	1	ref.			1	ref.		
	A few times a month	0.591	0.324	1.078	0.086	0.678	0.334	1.376	0.282
	One's a week or more often	0.721	0.388	1.34	0.301	0.524	0.248	1.105	0.09
Red meat, ham, sausages	A few times a month or less	1	ref.			1	ref.		
	2-4 times a week	1.682	1.082	2.614	0.021 *	1.898	1.135	3.174	0.015 *
	Every day	1.646	0.942	2.875	0.08	1.556	0.823	2.944	0.174
Sour milk products	A few times a month or less	1	ref.						
	2-4 times a week	1.098	0.67	1.799	0.71				
	Every day	1.101	0.635	1.91	0.731				

Variable		Univariate model				Multiple model			
		OR	95%CI		p	OR	95%CI		p
Cheese	A few times a month or less	1	ref.						
	2-4 times a week	1.275	0.809	2.01	0.296				
	Every day	1.403	0.835	2.358	0.202				
Cottage cheese	A few times a month or less	1	ref.			1	ref.		
	2-4 times a week	1.047	0.637	1.722	0.856	0.999	0.562	1.778	0.999
	Every day	2.54	1.356	4.756	0.004 *	1.978	0.963	4.062	0.063
Vegetables/fruit	Every day	1	ref.						
	Not every day	0.962	0.627	1.476	0.858				
Sweets/salty snacks	A few times a month or less	1	ref.						
	2-4 times a week	0.903	534	1.527	0.703				
	Every day	0.789	0.44	1.417	0.428				
Fast food products	I do not eat	1	ref.						
	I eat	0.785	0.526	1.171	0.236				

* Statistically significant relationship ($p<0.05$); *p* – single and multivariate linear regression; OR - odds ratio; CI – confidence interval; OR (95% CI) - odds ratio with a 95% confidence interval; WHtR - Waist to Height Ratio.

Table S-4. Consumption of specific group of products and the prevalence of overweight according to BMI. Univariate and multiple regression models.

Variable		Univariate model				Multiple model			
		OR	95%CI		p	OR	95%CI		p
Sex	Female	1	ref.			1	ref.		

Variable		Univariate model				Multiple model			
		OR	95%CI		p	OR	95%CI		p
	Male	1.496	0.63	3.552	0.361	1.768	0.652	4.797	0.263
Age	[years]	1.067	1.044	1.089	<0.001 *	1.066	1.042	1.091	<0.001 *
Adding sugar to coffe/tea	No	1	ref.						
	Yes	1.167	0.782	1.74	0.45				
Salting dishes	Rarely or never add salt to food	1	ref.						
	I taste the food and add salt as needed	0.87	0.514	1.473	0.605				
	I add salt to my food without trying it first	1.028	0.554	1.909	0.929				
White bread	Every day	1	ref.			1	ref.		
	Not every day	0.751	0.504	1.12	0.161	0.78	0.471	1.291	0.333
Wholemeal bread	A few times a month or less	1	ref.			1	ref.		
	2-4 times a week	1.373	0.867	2.174	0.176	1.543	0.909	2.621	0.108
	Every day	1.561	0.923	2.64	0.097	1.714	0.889	3.304	0.108
Fishes and seafood	I do not eat	1	ref.			1	ref.		
	A few times a month	0.442	0.238	0.822	0.01 *	0.44	0.219	0.882	0.021 *
	One’s a week or more often	0.824	0.432	1.572	0.557	0.597	0.284	1.255	0.174
Red meat, ham, sausages	A few times a month or less	1	ref.			1	ref.		
	2-4 times a week	2.184	1.394	3.423	0.001 *	2.208	1.347	3.618	0.002 *
	Every day	1.602	0.917	2.797	0.098	1.447	0.776	2.698	0.245
Sour milk products	A few times a month or less	1	ref.						
	2-4 times a week	0.918	0.556	1.516	0.739				
	Every day	0.945	0.541	1.653	0.844				

Variable		Univariate model				Multiple model			
		OR	95%CI		p	OR	95%CI		p
Cheese	A few times a month or less	1	ref.						
	2-4 times a week	1.005	0.636	1.589	0.982				
	Every day	1.363	0.803	2.315	0.251				
Cottage cheese	A few times a month or less	1	ref.			1	ref.		
	2-4 times a week	1.109	0.672	1.83	0.687	1.049	0.595	1.849	0.868
	Every day	1.617	0.877	2.983	0.124	1.128	0.566	2.248	0.732
Vegetables/fruit	Every day	1	ref.						
	Not every day	1.119	0.724	1.729	0.612				
Sweets/salty snacks	A few times a month or less	1	ref.						
	2-4 times a week	0.959	0.566	1.627	0.878				
	Every day	0.932	0.517	1.682	0.815				
Fast food products	I do not eat	1	ref.						
	I eat	1.068	0.714	1.596	0.75				

* Statistically significant relationship (p<0.05); *p* – single and multivariate linear regression; OR - odds ratio; CI – confidence interval; OR (95% CI) - odds ratio with a 95% confidence interval; BMI- Body Mass Index.

Table S-5. Consumption of specific group of products and the prevalence of obesity according to BMI. Univariate and multiple regression models.

Variable		Univariate model				Multiple model			
		OR	95%CI		p	OR	95%CI		p
Sex	Female	1	ref.			1	ref.		

Variable		Univariate model				Multiple model			
		OR	95%CI		p	OR	95%CI		p
	Male	0.505	0.169	1.508	0.221	0.506	0.156	1.646	0.258
Age	[years]	1.091	1.06	1.124	<0.001 *	1.095	1.061	1.129	<0.001 *
Adding sugar to coffe/tea	No	1	ref.			1	ref.		
	Yes	2.082	1.303	3.326	0.002 *	1.978	1.19	3.289	0.009 *
Salting dishes	Rarely or never add salt to food	1	ref.			1	ref.		
	I taste the food and add salt as needed	1.556	0.826	2.93	0.171	1.449	0.736	2.851	0.283
	I add salt to my food without trying it first	1.844	0.903	3.765	0.093	1.633	0.743	3.59	0.222
White bread	Every day	1	ref.						
	Not every day	0.764	0.487	1.197	0.239				
Wholemeal bread	A few times a month or less	1	ref.						
	2-4 times a week	1.014	0.608	1.693	0.957				
	Every day	0.977	0.546	1.748	0.937				
Fishes and seafood	I do not eat	1	ref.			1	ref.		
	A few times a month	0.541	0.291	1.006	0.052	0.612	0.308	1.216	0.161
	One’s a week or more often	0.617	0.326	1.167	0.137	0.598	0.297	1.205	0.15
Red meat, ham, sausages	A few times a month or less	1	ref.						
	2-4 times a week	1.199	0.73	1.969	0.474				
	Every day	1.126	0.602	2.105	0.711				
Sour milk products	A few times a month or less	1	ref.						
	2-4 times a week	1.006	0.581	1.741	0.983				
	Every day	0.821	0.439	1.535	0.537				

Variable		Univariate model				Multiple model			
		OR	95%CI		p	OR	95%CI		p
Cheese	A few times a month or less	1	ref.			1	ref.		
	2-4 times a week	1.467	0.868	2.479	0.152	1.439	0.812	2.55	0.212
	Every day	1.404	0.777	2.535	0.261	1.257	0.657	2.406	0.49
Cottage cheese	A few times a month or less	1	ref.						
	2-4 times a week	0.791	0.454	1.377	0.406				
	Every day	0.975	0.508	1872	0.939				
Vegetables/fruit	Every day	1	ref.						
	Not every day	1.247	0.777	2.001	0.36				
Sweets/salty snacks	A few times a month or less	1	ref.						
	2-4 times a week	1.14	0.627	2.073	0.668				
	Every day	1.203	0.62	2.335	0.584				
Fast food products	I do not eat	1	ref.						
	I eat	1.043	0.666	1.631	0.855				

* Statistically significant relationship (p<0.05); *p* – single and multivariate linear regression; OR - odds ratio; CI – confidence interval; OR (95% CI) - odds ratio with a 95% confidence interval; BMI- Body Mass Index.

Table S-6. Consumption of specific group of products and the prevalence of increased body fat. Univariate and multiple regression models.

Variable		Univariate model				Multiple model			
		OR	95%CI		p	OR	95%CI		p
Sex	Female	1	ref.			1	ref.		

Variable		Univariate model				Multiple model			
		OR	95%CI		p	OR	95%CI		p
	Male	2.131	0.933	4.866	0.073	3.296	1.281	8.478	0.013 *
Age	[years]	1.088	1.061	1.115	<0.001 *	1.093	1.065	1.121	<0.001 *
Adding sugar to coffe/tea	No	1	ref.						
	Yes	1.246	0.836	1.857	0.28				
Salting dishes	Rarely or never add salt to food	1	ref.						
	I taste the food and add salt as needed	0.765	0.455	1.284	0.31				
	I add salt to my food without trying it first	0.92	0.502	1.684	0.787				
White bread	Every day	1	ref.						
	Not every day	0.842	0.566	1.254	0.398				
Wholemeal bread	A few times a month or less	1	ref.			1	ref.		
	2-4 times a week	1.399	0.882	2.218	0.154	1.678	0.993	2.836	0.053
	Every day	1.099	0.651	1.856	0.724	1.177	0.653	2.12	0.587
Fishes and seafood	I do not eat	1	ref.			1	ref.		
	A few times a month	0.664	0.371	1.188	0.168	0.732	0.387	1.386	0.338
	One’s a week or more often	0.747	0.41	1.361	0.341	0.615	0.314	1.204	0.156
Red meat, ham, sausages	A few times a month or less	1	ref.						
	2-4 times a week	1.257	0.808	1.956	0.311				
	Every day	1.055	0.603	1.848	0.85				
Sour milk products	A few times a month or less	1	ref.						
	2-4 times a week	0.804	0.49	1.319	0.388				
	Every day	0.908	0.524	1.575	0.731				

Variable		Univariate model				Multiple model			
		OR	95%CI		p	OR	95%CI		p
Cheese	A few times a month or less	1	ref.						
	2-4 times a week	0.874	0.553	1.384	0.567				
	Every day	1.072	0.639	1.797	0.792				
Cottage cheese	A few times a month or less	1	ref.						
	2-4 times a week	0.987	0.596	1.637	0.961				
	Every day	1.307	0.719	2.377	0.38				
Vegetables/fruit	Every day	1	ref.						
	Not every day	0.891	0.578	1.372	0.6				
Sweets/salty snacks	A few times a month or less	1	ref.						
	2-4 times a week	0.861	0.511	1.451	0.574				
	Every day	0.936	0.522	1.677	0.824				
Fast food products	I do not eat	1	ref.			1	ref.		
	I eat	0.728	0.488	1.085	0.119	1.074	0.687	1.679	0.755

* Statistically significant relationship (p<0.05); *p* – single and multivariate linear regression; OR - odds ratio; CI – confidence interval; OR (95% CI) - odds ratio with a 95% confidence interval.

Table S-7. Consumption of specific group of products and the prevalence of and increased cardiovascular risk - Framingham Risc Score. Univariate and multiple regression models.

Variable		Univariate model				Multiple model			
		OR	95%CI		p	OR	95%CI		p
Sex	Female	1	ref.			1	ref.		
	Male	3.134	1.369	7.177	0.007 *	126.311	11.981	1331.671	<0.001 *

Variable		Univariate model				Multiple model			
		OR	95%CI		p	OR	95%CI		p
Age	[years]	1.623	1.455	1.811	<0.001 *	1.751	1.535	1.997	<0.001 *
Adding sugar to coffe/tea	No	1	ref.						
	Yes	1.177	0.776	1.786	0.443				
Salting dishes	Rarely or never add salt to food	1	ref.						
	I taste the food and add salt as needed	0.71	0.416	1.213	0.21				
	I add salt to my food without trying it first	0.929	0.5	1.728	0.817				
White bread	Every day	1	ref.			1	ref.		
	Not every day	0.571	0.373	0.872	0.01 *	1.016	0.473	2.179	0.968
Wholemeal bread	A few times a month or less	1	ref.						
	2-4 times a week	0.787	0.489	1.266	0.324				
	Every day	0.776	0.452	1.333	0.359				
Fishes and seafood	I do not eat	1	ref.			1	ref.		
	A few times a month	0.613	0.336	1.12	0.112	0.809	0.295	2.215	0.68
	One's a week or more often	0.926	0.502	1.706	0.804	0.793	0.288	2.185	0.653
Red meat, ham, sausages	A few times a month or less	1	ref.			1	ref.		
	2-4 times a week	1.455	0.911	2.325	0.116	1.619	0.712	3.679	0.25
	Every day	1.466	0.819	2.624	0.198	1.551	0.572	4.206	0.389
Sour milk products	A few times a month or less	1	ref.			1	ref.		
	2-4 times a week	0.654	0.391	1.093	0.105	0.722	0.302	1.727	0.464
	Every day	0.911	0.519	1.6	0.746	0.654	0.239	1.788	0.408
Cheese	A few times a month or less	1	ref.						

Variable		Univariate model				Multiple model			
		OR	95%CI		p	OR	95%CI		p
	2-4 times a week	0.955	0.593	1.54	0.851				
	Every day	0.989	0.576	1.698	0.969				
Cottage cheese	A few times a month or less	1	ref.			1	ref.		
	2-4 times a week	1.029	0.596	1.774	0.919	0.7	0.272	1.803	0.46
	Every day	2.296	1.231	4.282	0.009 *	2.058	0.685	6.179	0.198
Vegetables/fruit	Every day	1	ref.						
	Not every day	0.889	0.565	1.398	0.61				
Sweets/salty snacks	A few times a month or less	1	ref.						
	2-4 times a week	0.767	0.448	1.314	0.334				
	Every day	0.833	0.456	1.52	0.551				
Fast food products	I do not eat	1	ref.			1	ref.		
	I eat	0.705	0.466	1.068	0.099	1.919	0.922	3.993	0.081

* Statistically significant relationship ($p<0.05$); *p* – single and multivariate linear regression; OR - odds ratio; CI – confidence interval; OR (95% CI) - odds ratio with a 95% confidence interval.
